# Bayesian Borrowing of External Information in Clinical Trials: A Comparison of MAP, RMAP, and SAM Priors

**DOI:** 10.64898/2026.08.26.26360843

**Published:** 2026-08-31

**Authors:** Leena Choi, Elizabeth McNeer, Cole A. Beck, Jeffrey L. Neul

## Abstract

Bayesian borrowing of external information can improve trial efficiency, particularly in pediatric and rare disease settings where patient populations are limited, but may introduce bias and inflate the Type I error rate when the trial differs from external studies. Recent U.S. Food and Drug Administration (FDA) draft Bayesian guidance emphasizes careful evaluation of external information, prior specification, and assessment of operating characteristics.

This paper compares three meta-analytic-predictive (MAP)-based methods for Bayesian borrowing: the MAP prior, robust MAP (RMAP) prior, and self-adapting mixture (SAM) prior. An adaptive platform trial design in Rett syndrome is used as a case study. Simulation studies evaluate frequentist operating characteristics under varying prior–data conflict, between-study heterogeneity, treatment effects, and clinically significant differences (CSDs) for the SAM prior.

The MAP prior achieved the greatest efficiency when external and current data were compatible but exhibited the largest bias under substantial prior–data conflict. The RMAP priors improved robustness through fixed robust-component weights, whereas the SAM prior adaptively adjusted borrowing and was less sensitive to prior–data conflict while retaining efficiency gains when the data were compatible. Although the CSD influenced the degree of adaptive borrowing, as reflected by effective sample size, it had only a modest impact on frequentist operating characteristics. Sensitivity analyses using a skeptical robust component yielded similar qualitative conclusions, while accentuating the differences between the MAP and RMAP priors.

These findings provide guidance for evaluating and selecting MAP-based borrowing strategies before trial implementation, particularly in rare disease settings, consistent with current FDA recommendations.

## Full Text Availability

The license terms selected by the author(s) for this preprint version do not permit archiving in PMC. The full text is available from the preprint server.

